# Distribution of Endogenous NO Regulates Early Gravitropic Response and PIN2 Localization in Arabidopsis Roots

**DOI:** 10.3389/fpls.2018.00495

**Published:** 2018-04-20

**Authors:** Ramiro París, María M. Vazquez, Magdalena Graziano, María C. Terrile, Nathan D. Miller, Edgar P. Spalding, Marisa S. Otegui, Claudia A. Casalongué

**Affiliations:** ^1^Instituto de Investigaciones Biológicas, UE Consejo Nacional de Investigaciones Científicas y Técnicas-UNMDP, Facultad de Ciencias Exactas y Naturales, Universidad Nacional de Mar del Plata, Mar del Plata, Argentina; ^2^Department of Botany, University of Wisconsin, Madison, WI, United States; ^3^Laboratory of Cell and Molecular Biology, Departments of Botany and Genetics, University of Wisconsin, Madison, WI, United States

**Keywords:** Arabidopsis, auxin, auxin transport, gravitropism, nitric oxide, root, tropic response

## Abstract

High-resolution and automated image analysis of individual roots demonstrated that endogenous nitric oxide (NO) contribute significantly to gravitropism of Arabidopsis roots. Lowering of endogenous NO concentrations strongly reduced and even reversed gravitropism, resulting in upward bending, without affecting root growth rate. Notably, the asymmetric accumulation of NO along the upper and lower sides of roots correlated with a positive gravitropic response. Detection of NO by the specific DAF-FM DA fluorescent probe revealed that NO was higher at the lower side of horizontally-oriented roots returning to initial values 2 h after the onset of gravistimulation. We demonstrate that NO promotes plasma membrane re-localization of PIN2 in epidermal cells, which is required during the early root gravitropic response. The dynamic and asymmetric localization of both auxin and NO is critical to regulate auxin polar transport during gravitropism. Our results collectively suggest that, although auxin and NO crosstalk occurs at different levels of regulation, they converge in the regulation of PIN2 membrane trafficking in gravistimulated roots, supporting the notion that a temporally and spatially coordinated network of signal molecules could participate in the early phases of auxin polar transport during gravitropism.

## Introduction

Plants maintain developmental continuity and adapt their growth and differentiation to the constantly changing environment by redefining cell polarities. At the level of an individual cell, polarity is commonly associated with the asymmetric distribution of intracellular components such as proteins, plant hormones, and other signal molecules. A relevant case of a polarized-tropic response is the root reorientation toward the gravity vector or gravitropism, which enables plants to precisely orient their root system to anchor themselves and to explore the soil for water and nutrients (Blancaflor and Masson, 2003).

Arabidopsis root bending toward gravity is driven by formation of an auxin gradient between the upper and the lower sides of the root, which in turn, is established by cell-to-cell auxin polar transport during gravitropism (Leyser, 2005). This is a dynamic phenomenon that rapidly redistributes auxin to the lower side of the root within minutes of a gravity stimulus (Band et al., 2012). Directional auxin transport is highly regulated at the molecular level (Spalding, 2013), by plasma-membrane resident influx and efflux carriers such as Auxin-Resistant 1 (AUX1) and ATP-Binding Cassette B-type (ABCB) proteins, and efflux facilitators PIN-Formed (PIN) proteins (Friml, 2010; Zazímalová et al., 2010).

Auxin efflux facilitators of the Arabidopsis PIN family show a dynamic polar localization in the root cells as they control the direction of auxin flow during gravitropism (Blakeslee et al., 2005; Paponov et al., 2005). In general, some members of the Arabidopsis PIN family show a dynamic polar localization, which is mediated by regulatory mechanisms including endocytocis and recycling through vesicle trafficking (Grunewald and Friml, 2010; Reynolds et al., 2018). Auxin itself affects PIN2 trafficking and turnover in the root (Abas et al., 2006; Pan et al., 2009).

During gravitropism one of the main players that allow the transport of auxin from the tip to the elongation zone in Arabidopsis roots is PIN2 (Rahman et al., 2010). PIN2 is localized in the shootward-facing membranes of lateral root cap (LRC), epidermal, and mature cortical cells (Müller et al., 1998; Abas et al., 2006). Hence, PIN2-mediated asymmetric distribution of auxin leads to a differential growth between the lower and the upper side of the root. Thus, the mechanisms underlying the spatiotemporal asymmetric distribution of PIN2 and auxin are critical to understand growth-tropic responses in plants (Grunewald and Friml, 2010).

Besides auxin, sedimentation of starch-filled amyloplasts within the columella cells of the root cap constitutes one of the initial events in gravity perception (Blancaflor and Masson, 2003). The glutamate receptors controlling Ca^2+^ influx and LAZY plant-specific proteins are early-acting mediators of gravity signals (Miller et al., 2010; Taniguchi et al., 2017; Yoshihara and Spalding, 2017). In addition, apoplastic pH, Ca^2+^, inositol 1,4,5-trisphosphate, and reactive oxygen species have been associated to root gravitropism (Perera et al., 1999; Scott and Allen, 1999; Joo et al., 2001; Monshausen et al., 2011). It has been proposed that all these signals together, act in the initial non-transcriptional phase of the gravitropic response, within 15 min after the onset of the gravistimulus (Sato et al., 2015). In gravistimulated soybean roots, the auxin transport inhibitor 1-N-naphthylphthalamic acid impaired nitric oxide (NO) accumulation suggesting that NO is a downstream effector of auxin action (Hu et al., 2005). However, whether there is a direct crosstalk between auxin and NO in root gravitropism is currently unknown. To shed light on the molecular basis that underpins auxin and NO regulation, we analyzed gravistimulated Arabidopsis roots using an accurate, time-resolved morphometric analysis and quantitative confocal imaging. We also analyzed the asymmetric distribution of auxin and NO and discussed their functions on regulating PIN2 dynamics during the initial phases of root gravitropism in Arabidopsis.

## Materials and methods

### Plant material and growth conditions

*Arabidopsis thaliana* mutant strains and transgenic lines reported herein are in Col-0 background. The *35S::PIN2-GFP* and *PIN2::PIN2-GFP* transgenic lines, previously described by Abas et al. (2006) were obtained from Arabidopsis Biological Resource Center at Ohio State University, USA; *sav3*-3 were described in Tao et al. (2008). *yuc*Q (*yuc3/5/7/8/9*) reported in Li et al. (2012) and *tir1*-1/*afb2*-3 in Calderón Villalobos et al. (2012). The seeds used in all experiments were passed through a U.S. standard brass test sieve (Fisher Scientific Co., USA) grading sizes of 280 μm; only seeds retained on the sieve were used. Seeds were surface-sterilized with 70% (v/v) ethanol for 10 min, 35% (v/v) bleach for 1 min and rinsed five times with sterile water. Seeds were placed on petri dishes containing 0.5 × Murashige and Skoog salt, 0.8% (w/v) agar and supplemented with 1% (w/v) sucrose, stratified at 4°C for 3 days in the dark, and set to germinate. Petri dishes were placed vertically in a growth chamber under controlled conditions 23°C, light 120 μmol photons m^−2^ s^−1^, with a 16:8 h light:dark cycle.

### Chemicals

Stock solutions of both, the NO scavenger 2-(4-carboxyphenyl)-4,4,5,5-tetramethylimidazoline-1-oxy-3-oxid, potassium salt (cPTIO, Molecular Probes, USA) and NO donor S-nitroso-L-cysteine (CysNO) were prepared as previously described by Martínez-Ruiz and Lamas (2007). 1-Naphtalenacetic acid (NAA) was from BDH, England, Brefeldin A (BFA) and Cycloheximide (CHX) were from Sigma-Adrich, USA. NAA, BFA, and CHX chemical solutions were prepared as previously described by Wang et al. (2013). Stock solutions were prepared at: 50 mM for CHX and BFA, 10 mM for NAA, 100 mM for cPTIO, and 50 mM for CysNO (concentration before use was determined from absorbance at 338 nm ε_338_ = 900 M^−1^ cm^−1^). Unless otherwise indicated, final working solutions were 50 μM for CHX and BFA and 10 μM for NAA. Pretreatments with CHX, cPTIO, CysNO, and NAA were for 30 min, treatments with BFA were for 60 min, any other pretreatment and treatment time are indicated in the text. In controls, equal amounts of solvent were used.

### Root gravitropic assay

After 3 days of growth, seedlings were transferred to fresh 0.5 × MS medium 0.8% (w/v) agar and supplemented with 1% (w/v) sucrose containing different concentrations of cPTIO or CysNO. Solutions were also poured at the surface of each root to ensure homogeneous absorption and action. The plates were mounted vertically and transverse to the optical axes of CCD cameras and let set for 60 min (Durham Brooks et al., 2010). After root gravistimulation, images were taken every 2 min for 8 h at 100 px/mm using infrared-sensitive CCD cameras. For measuring root growth parameters and tip angle, a morphometric image-processing algorithm developed by Miller et al. (2007) was used.

### Confocal microscopy and image analysis

For the detection of endogenous NO, 3-day-old seedlings were gravistimulated by 90° rotation and loaded with a 10 μM solution of the cell-permeable fluorescent probe 3-amino, 4-aminomethyl-2,7-difluorofluorescein diacetate (DAF-FM DA, Molecular Probes, USA) in 5 mM MES pH 5.7 containing 0.25 mM KCl and 1 mM CaCl_2_ for 30 min. Before observation, seedlings were washed 3 times with the same buffer. Finally, DAF-FM DA-dependent fluorescence was detected in a confocal microscope (Nikon Eclipse C1 Plus) using a 20 x objective, NA = 0.75, an argon ion laser set at 488 nm; emission was collected between 500 and 530 nm. Six z-stacks were collected at 6 μm axial interval. Single pictures with no saturated pixels were combined into a sum of slices projection with the aid of FIJI bundle software (Schindelin et al., 2012). The fluorescence intensity of each individual root was quantified on digital images within independent regions of interests located in the lower and upper side of the epidermal root cells and in the outermost layer of the cortical root cells by using FIJI (Schindelin et al., 2012).

For the detection of PIN2-GFP, 3-day-old seedlings were gravistimulated by 90° rotation. Fluorescence was detected by confocal microscopy. Images were obtained with a Nikon Eclipse C1 Plus, 40 x objective, NA = 1.3 with an argon ion laser set at 488 nm; emission was collected between 500 and 530 nm. To obtain images under gravitropic stimulus we used a Zeiss LSM333 confocal microscope equipped with a vertical stage (InverterScope®) 20 x objective, NA = 0.8 with an argon ion laser set at 488 nm; emission was collected between 490 and 561 nm. Fluorescence intensity at the plasma membrane was quantified in the shootward and rootward sides of root cells from digital images with no saturated pixels using FIJI (Schindelin et al., 2012).

## Results

### Endogenous NO is necessary during early gravitropism in arabidopsis roots

To study the function of NO signal during root gravitropism, we first analyzed individual Arabidopsis roots treated with different chemicals to modulate endogenous NO levels and incubated with the DAF-FM DA probe that becomes fluorescent upon reacting with NO. As expected, treatment with the NO donor CysNO resulted in an increase of DAF-FM DA fluorescent signal within roots, whereas the NO-scavenger cPTIO efficiently reduced the fluorescent signal to ~30% of the control roots (Figures S1A,B).

**Figure 1:**
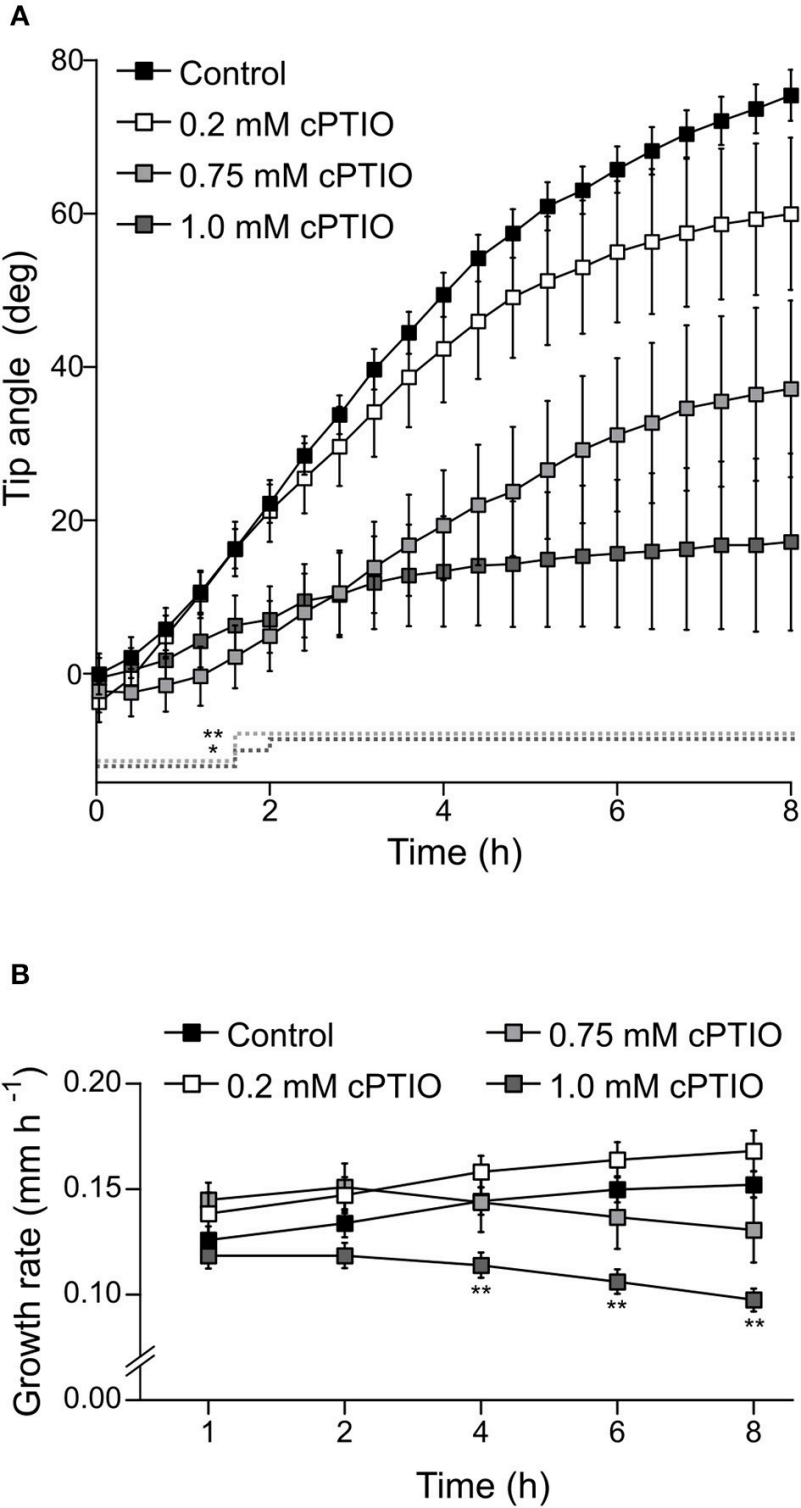
Tip angle in NO-depleted roots after gravistimulation. Arabidopsis seedlings were vertically grown for 3 days and then treated with the indicated concentrations of cPTIO as NO scavenger or water as control for 60 min before being rotated by 90°. Roots were monitored every 2 min for 8 h. **(A)** Root tip angle was determined by a software-based analysis as indicated in section Materials and Methods. Error bars representing SE are indicated for 24 min intervals. *P*-values are represented by dashed lines as upward deflections. No statistically significant differences were found between control and 0.2 mM cPTIO treatment. **(B)** Average values (±SE) of growth rate expressed as mm per h. The average values of at least 4 independent experiments were analyzed, *n* ≥ 16. *P*-values in comparison to control were calculated with two-tailed Student's *t*-test, ^**^P ≤ 0.01, ^*^P ≤ 0.05.

**Figure 2:**
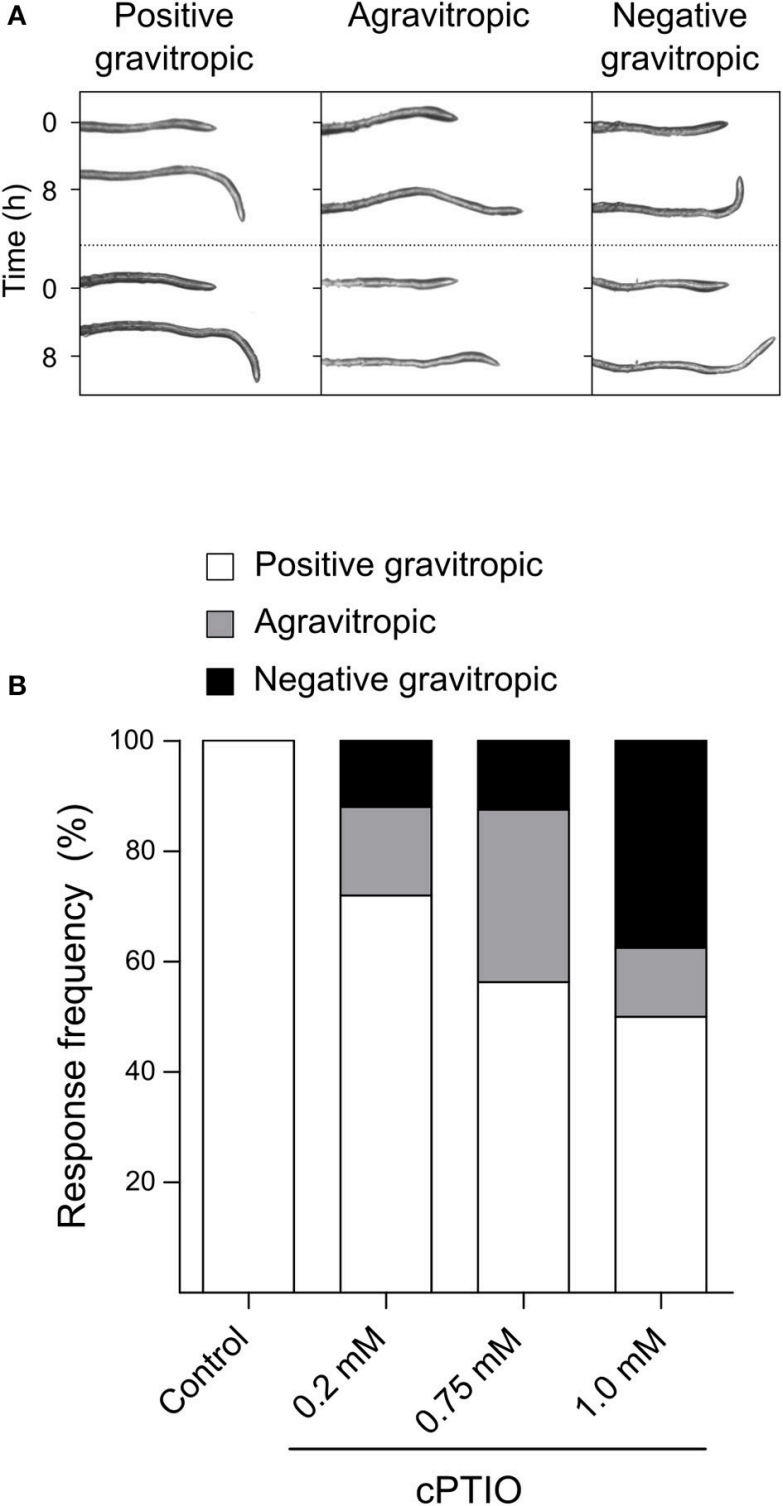
Frequency distribution of gravitropic responses in NO depleted roots. Arabidopsis seedlings were grown and incubated with cPTIO as indicated in Figure 1. At 8 h after 90° gravistimulation, values of tip angles were recorded and classified in three categories depending on the root tip angle: agravitropic (−10° to 30°), negatively gravitropic (< −10°) and positively gravitropic roots (>30°). **(A)** Two representative roots of each category are shown. **(B)** Frequency distribution of root categories measured in control conditions and under 0.2, 0.75, and 1 mM cPTIO treatments. At least 4 independent experiments were performed, *n* ≥ 16.

**Figure 3:**
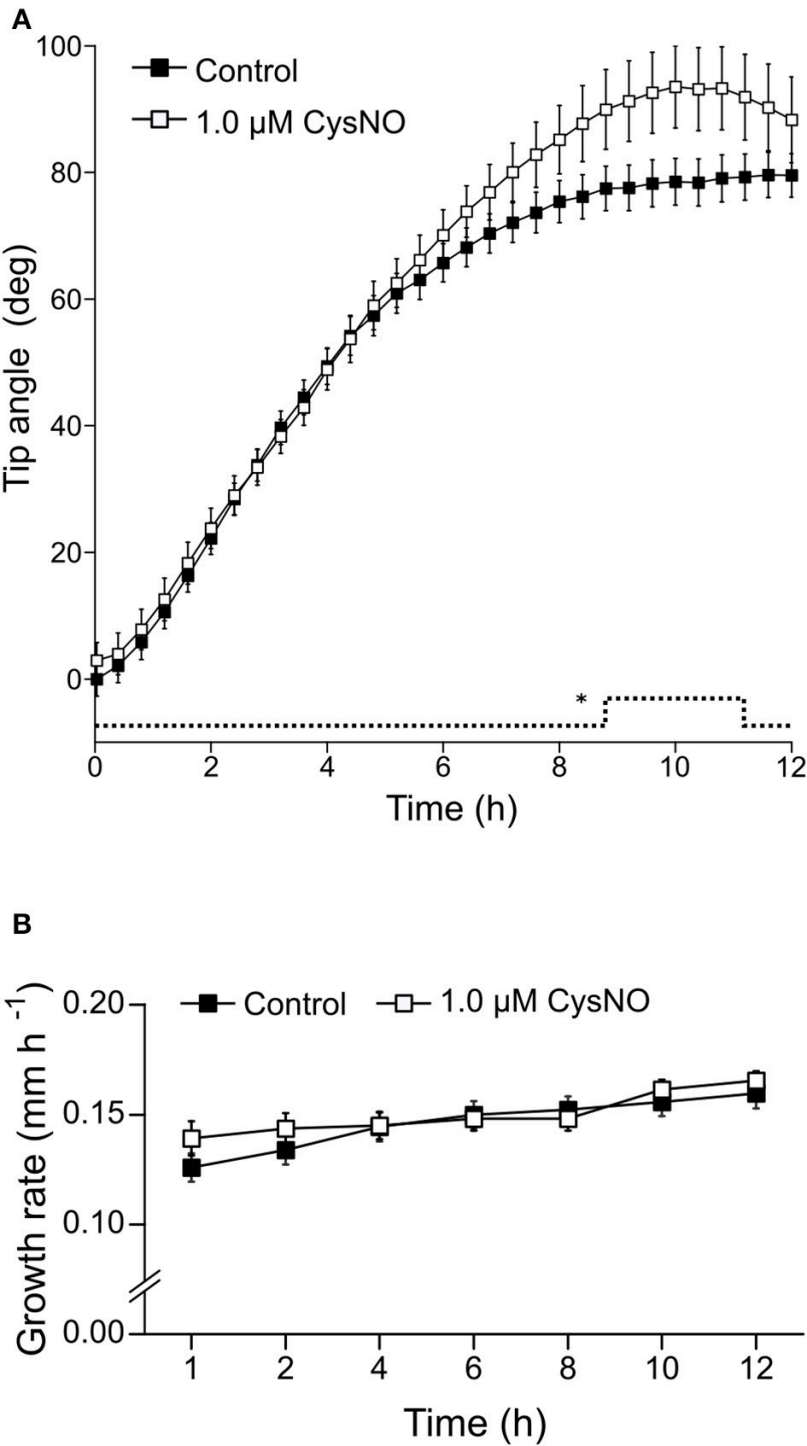
Tip angle in CysNO-treated and gravistimulated roots. Arabidopsis seedlings were vertically grown for 3 days and then treated with 1.0 μM CysNO or water for 60 min before being rotated by 90°. Roots were monitored every 2 min during 12 h. **(A)** Average values of tip angles were determined by a software-based analysis as indicated in section Materials and Methods. Error bars representing SE are indicated for 24 min-intervals. *P*-values are represented by dashed lines as upward deflections. **(B)** Average values (±SE) of growth rate, expressed as mm per h. At least 3 independent experiments were performed, *n* ≥ 11. *P*-values in comparison to control were calculated with two-tailed Student's *t*-test, ^*^P ≤ 0.05.

Once we established that these two drugs effectively modulate NO concentration within roots, we investigated whether they affect root gravitropic responses in Arabidopsis. We found that cPTIO-treated roots showed a wide variation tip curvature 8 h after gravistimulation (Figure 1A). Moreover, the reduction in the tip angle in cPTIO-treated roots was statistically significant as early as 1.5 h after gravistimulation. The average values of tip angles at the last assayed time (8 h after gravistimulation), were 75.4, 59.9, 37.1, and 17.2° for untreated, 0.2, 0.75, and 1.0 mM cPTIO-treated roots, respectively. Compared to control, roots treated with 0.2 and 0.75 mM cPTIO did not show changes in their growth rates; however, roots treated with 1.0 mM cPTIO showed reduced growth rate starting at 4 h after gravistimulation (Figure 1B). By analyzing individual roots (Figure S2), we identified 3 types of gravitropic patterns at 8 h after gravistimulation: (i) agravitropic roots with −10° to 30° tip angles, (ii) roots that responded negatively to gravity (root tip angles <-10°), or (iii) roots that responded positively to gravity (root tip angles >30°; Figure 2A). Representative roots displaying each pattern are shown in supplementary movies (Movies S1–S4). Figure 2B shows the frequency distribution of root tip angle belonging to each category. At 0.2 mM cPTIO 72% of roots were positively gravitropic, 12% were agravitropic, and 16% were negatively gravitropic. At 0.75 and 1.0 mM cPTIO, the frequency of atypical gravitropic responses (agravitropic and negatively gravitropic) raised to 40 and 50%, respectively. On the other hand, 1.0 μM CysNO-treated roots showed wider bending angles than controls starting at 6 h after gravistimulation, leading to a transient overshooting with a maximum average root tip angle of 93.5° at 10 h after gravistimulation (Figure 3A). CysNO applied at 1.0 μM did not exert effect on growth rate within 12 h after onset of gravistimulation (Figure 3B).

### NO modulates auxin-dependent PIN2 redistribution in gravistimulated roots

**Figure 4:**
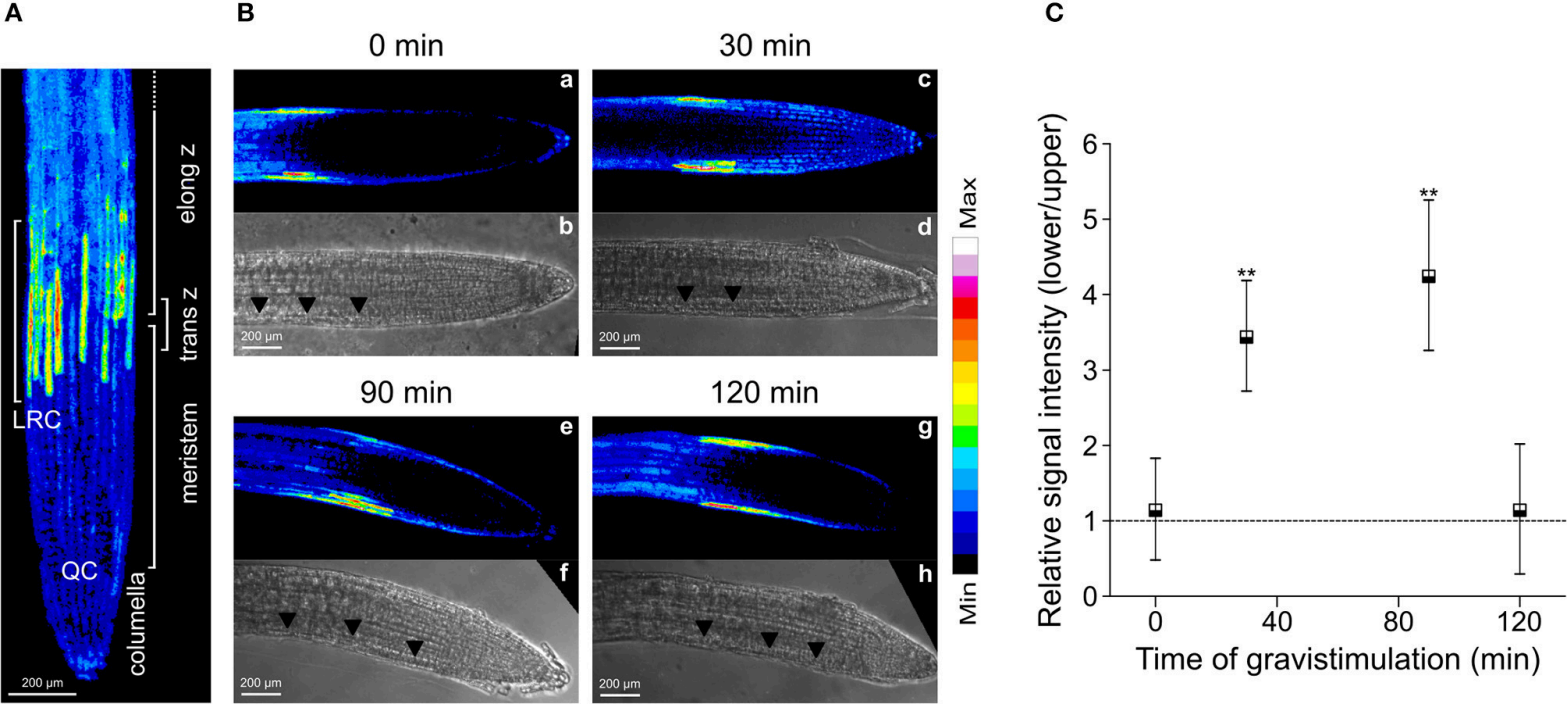
Distribution of NO in gravistimulated Arabidopsis roots. Three-day-old seedlings were pre-loaded with the NO-specific fluorescent probe DAF-FM DA. Accumulation of NO is shown as false colors in **(A)** maximal intensity z projection of root sections (nine z-sections spaced ~6 μm), lateral root cells (LRC), quiescent center (QC), columella, meristem, transition zone (trans z), and elongation zone (elong z) are indicated; **(B)** medial optical sections of representative roots at 0; 30; 90 and 120 min after 90° gravistimulation **(a,c,e,g)**. Signal intensities are coded black to white corresponding to their intensity. Bright field images **(b,d,f,h)**, arrowheads indicate the epidermal cells at the lower side in the root. **(C)** Quantification of DAF-FM DA-mediated fluorescence signal on lateral root cap and epidermal cells. Fluorescence signal intensity ratio between the lower and upper sides of the root at different times after gravistimulation. Means (±SE) of at least 3 independent experiments were analyzed, *n* ≥ 17. *P*-values in comparison to time 0 min were calculated with two-tailed Student's *t*-test, ^**^P ≤ 0.01.

**Figure 5:**
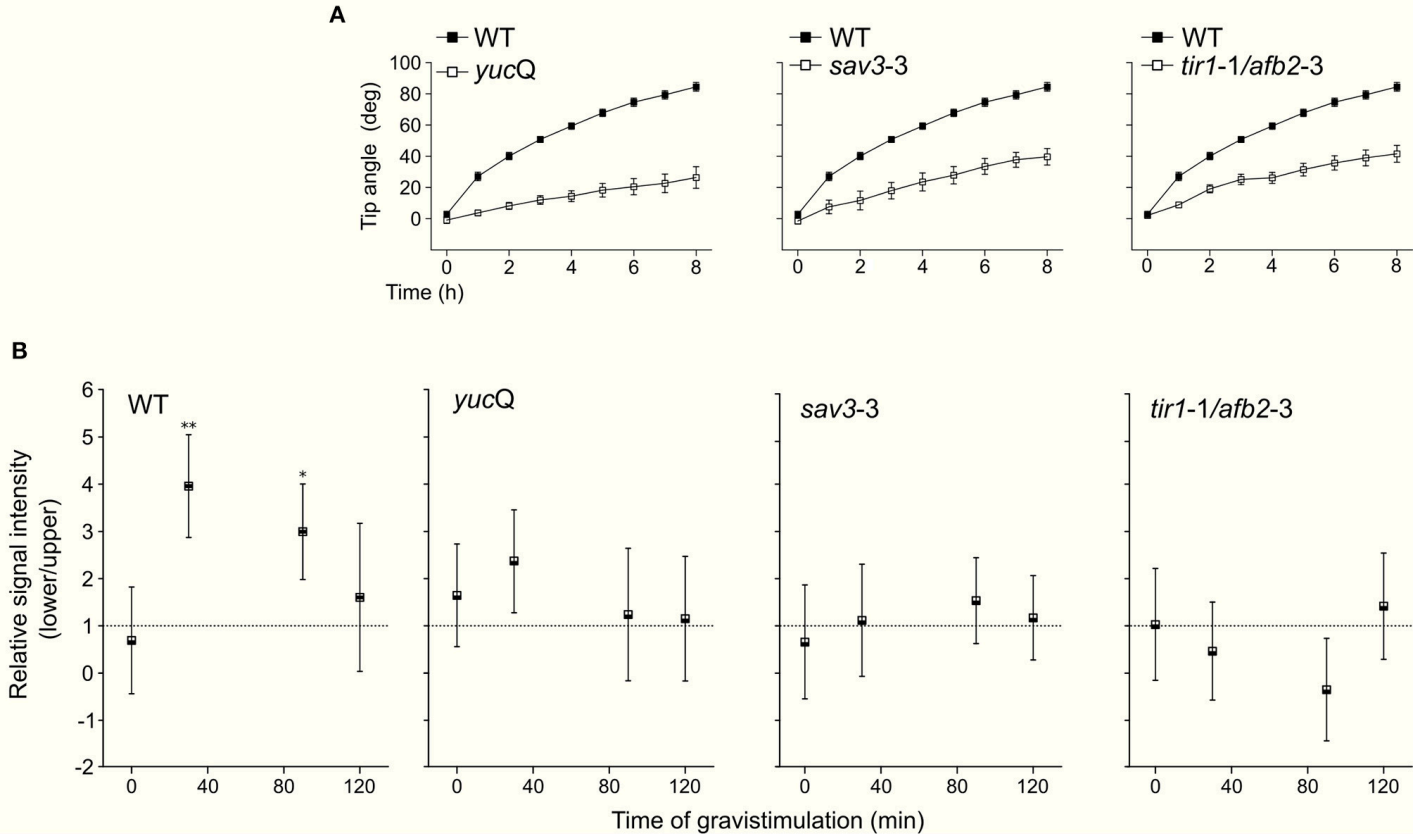
Tip angle and distribution of NO in gravistimulated mutant plants. Arabidopsis WT, *yuc*Q, *sav3*-3, and *tir1*-1/*afb2*-3 seedlings were vertically grown for 3 days and then rotated by 90°. Roots were monitored every 60 min during 8 h. **(A)** Average values of tip angles. Error bars representing SE are indicated. **(B)** Quantification of DAF-FM DA-mediated fluorescence signal on lateral root cap and epidermal cells. Fluorescence signal intensity ratio between the lower and upper sides of the root at different times after gravistimulation. Means (±SE) of at least 3 independent experiments were analyzed, *n* ≥ 12. *P*-values in comparison to time 0 min were calculated with two-tailed Student's *t*-test, ^**^P ≤ 0.01, ^*^P ≤ 0.05.

PIN2 promotes asymmetric auxin distribution and accumulation in the lower side of the horizontally oriented roots (Sato et al., 2015). Thus, we hypothesized that auxin-mediated PIN2 redistribution in gravistimulated Arabidopsis roots could be dependent on asymmetric localization of endogenous NO. We quantified NO accumulation at the lower and upper sides of individual roots by imaging DAF-FM DA fluorescence. In non-gravistimulated roots, the DAF-FM DA fluorescent signal was symmetrically distributed along the root tip area. The strongest fluorescence signal was detected 1 mm from the root tip, in a region ~400 μm long, comprising epidermal and lateral root cap cells (Figure 4A). At 30 and 90 min after gravistimulation, DAF-FM DA signal became brighter and more extended at the lower side of the root, including the elongation and meristematic zones. However, no evident changes occurred along the same zones in the upper side of the roots (Figure 4B). Estimation of the fluorescence ratio between lower and upper sides of the root tip confirmed that NO became asymmetrically distributed; DAF-FM DA fluorescent signal was higher at the lower side within 30 and 90 min, but returned to initial values at 120 min after the onset of gravistimulation (Figure 4C). The *yuc*Q (Li et al., 2012) and *sav3*-3 (Stepanova et al., 2008; Tao et al., 2008) mutants, which are defective in the indole-3-pyruvic acid pathway and IAA synthesis, and the double *tir1*-1/*afb2*-3 mutant with impaired auxin signaling (Calderón Villalobos et al., 2012) showed significant defects in root gravitropic response (Figure 5A). Interestingly, these three mutants did not show the asymmetric pattern of NO distribution after gravitropic stimulation seen in wild type plants (Figure 5B).

**Figure 6:**
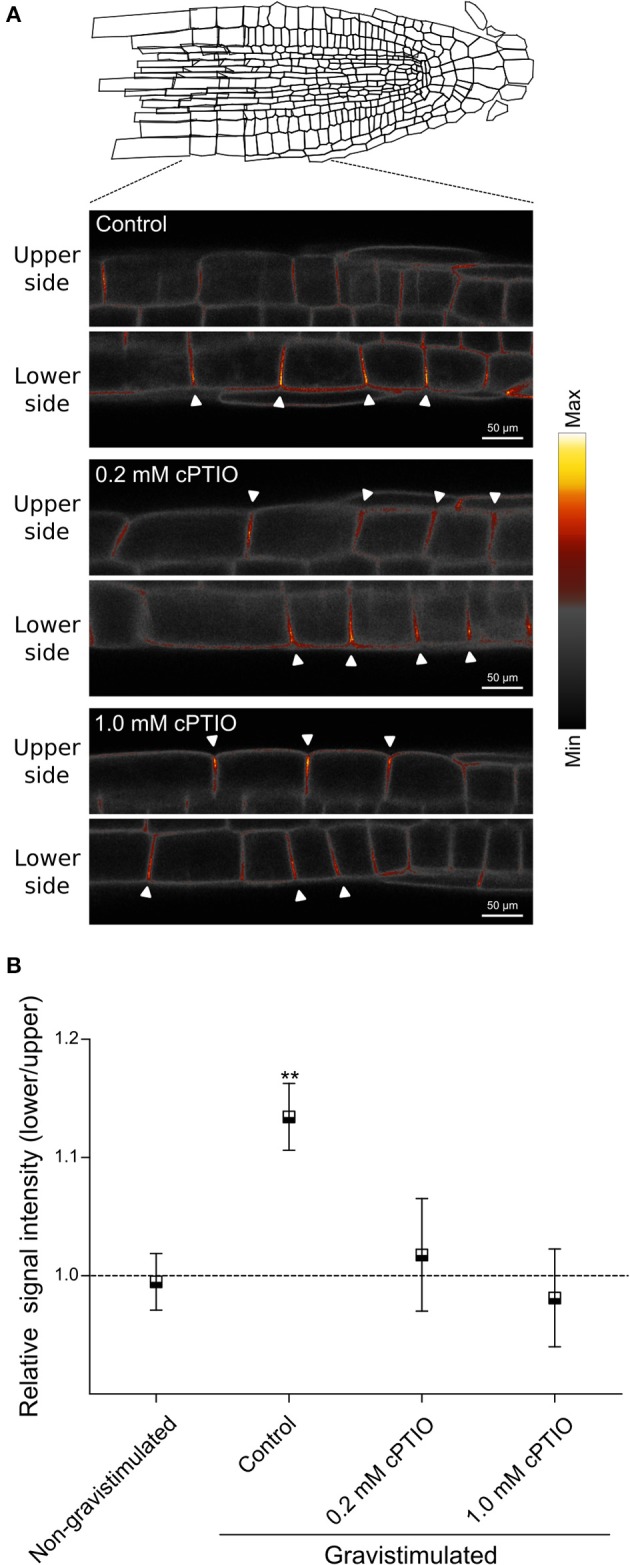
Plasma membrane localization of PIN2-GFP in NO-depleted roots during gravitropism. Arabidopsis seedlings were vertically grown for 3 days and then, treated with the indicated concentrations of cPTIO as NO scavenger, or water as control for 60 min before being rotated by 90°. **(A)** Localization of PIN2-GFP at the plasma membrane of epidermal root cells is shown as false colors in representative roots from control, 0.2 and 1.0 mM cPTIO treated seedlings at 120 min after gravistimulation. Arrowheads indicate PIN2-GFP fluorescence signal at the plasma membrane. **(B)** Ratio of PIN2-GFP signal intensity between lower and upper sides of gravistimulated roots. Means (±SE) of at least 3 independent experiments were analyzed, *n* ≥ 17. *P*-values in comparison to non-gravistimulated treatment calculated with two-tailed Student's *t*-test, ^**^P ≤ 0.01.

**Figure 7:**
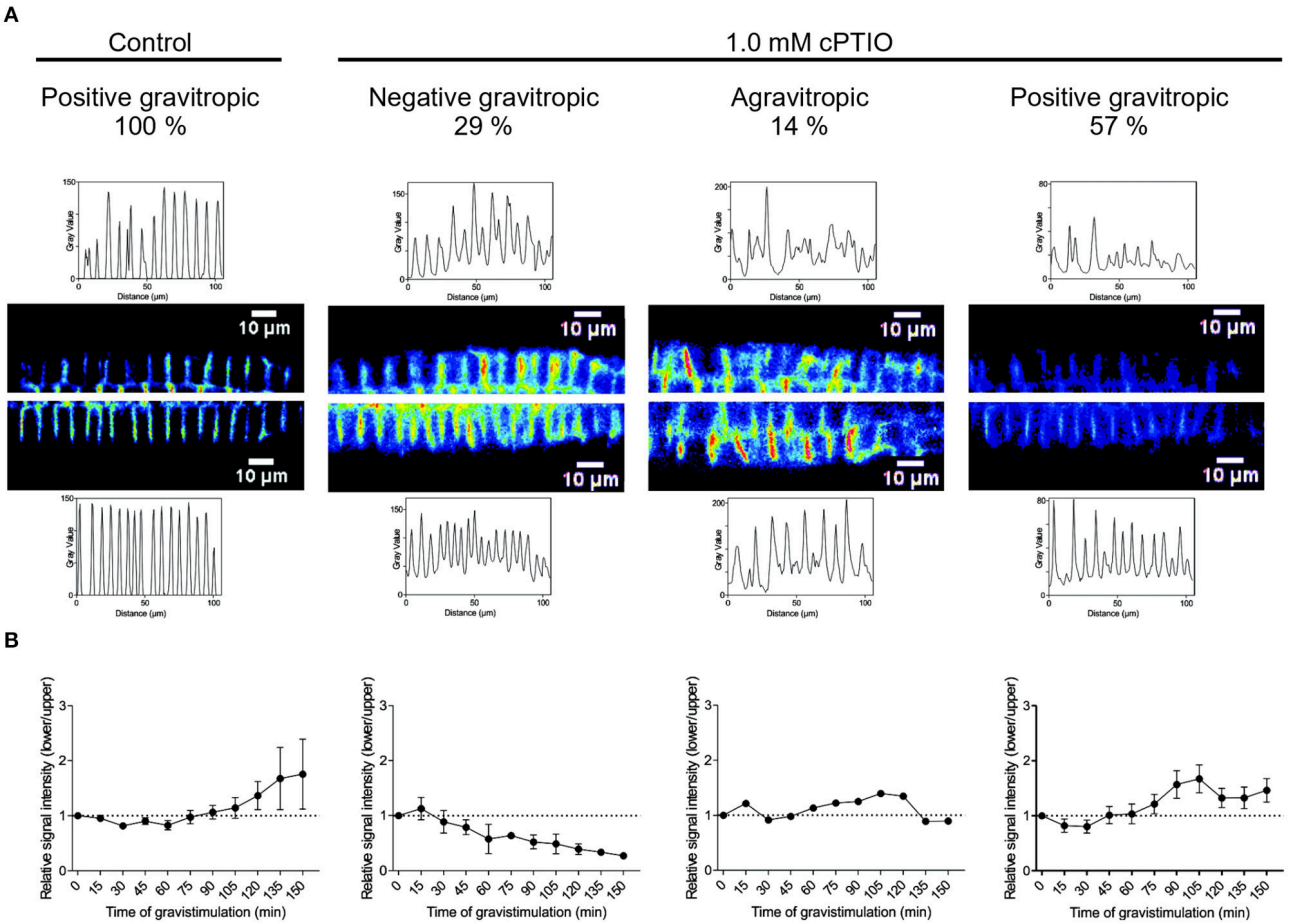
Localization of PIN2-GFP correlates with the gravitropic response in NO-depleted roots. Arabidopsis seedlings were vertically grown, treated as shown in Figure 6 and mounted in a vertical stage. Pictures were taken at 15 min intervals. After 120 min of gravistimulation, roots were classified in 3 categories depending on the root tip angle as: agravitropic, negatively gravitropic, and positively gravitropic roots. **(A)** Localization of PIN2-GFP signal intensity in the plasma membrane of epidermal root cells is shown in representative roots from control and 1.0 mM cPTIO-treated seedlings at 120 min after gravistimulation. **(B)** Ratio of PIN2-GFP signal intensity between lower and upper sides of gravistimulated roots. Means (±SE) of 2 independent experiments were analyzed, *n* ≥ 4.

To investigate whether PIN2 relocalization depends on NO availability, we studied PIN2 distribution at the plasma membrane in gravistimulated roots. As previously demonstrated by Baster et al. (2013), at 120 min after gravistimulation PIN2-GFP was asymmetrically distributed in the epidermal cells, showing a stronger signal along the lower than upper side of the root, returning promptly to the symmetric distribution at ~150 min (Figure S3). We then analyzed PIN2-GFP fluorescence distribution in cPTIO-treated roots at 120 min after gravistimulation. The ratio of PIN2-GFP signal in epidermal cells between the lower and upper root sides was quantified in roots treated with 0.2 and 1.0 mM cPTIO. Interestingly, under these two concentrations of cPTIO, gravistimulated roots showed no differences between upper/lower PIN2-GFP intensity ratios compared to those from non-gravistimulated roots (Figures 6A,B), which is consistent with the altered gravitropic responses shown in Figures 1, 2. To further study PIN2-GFP localization in NO-depleted seedlings, we collected confocal fluorescence images of single gravistimulated roots every 30 min in a time period of 150 min. Root tip angle was measured at these intervals of time and the PIN2-GFP signal ratio between lower and upper epidermal cell membranes was calculated. Notably, cPTIO-treated roots that displayed negative gravitropic responses showed an inverted PIN2 distribution pattern with increased PIN2-GFP fluorescence in the upper side (Figures 7A,B). Meanwhile, agravitropic roots were correlated to a rather symmetric PIN2-GFP distribution between lower and upper epidermal cells; and positive gravitropic roots showed PIN2-GFP distribution similar to controls (Figures 7A,B).

**Figure 8:**
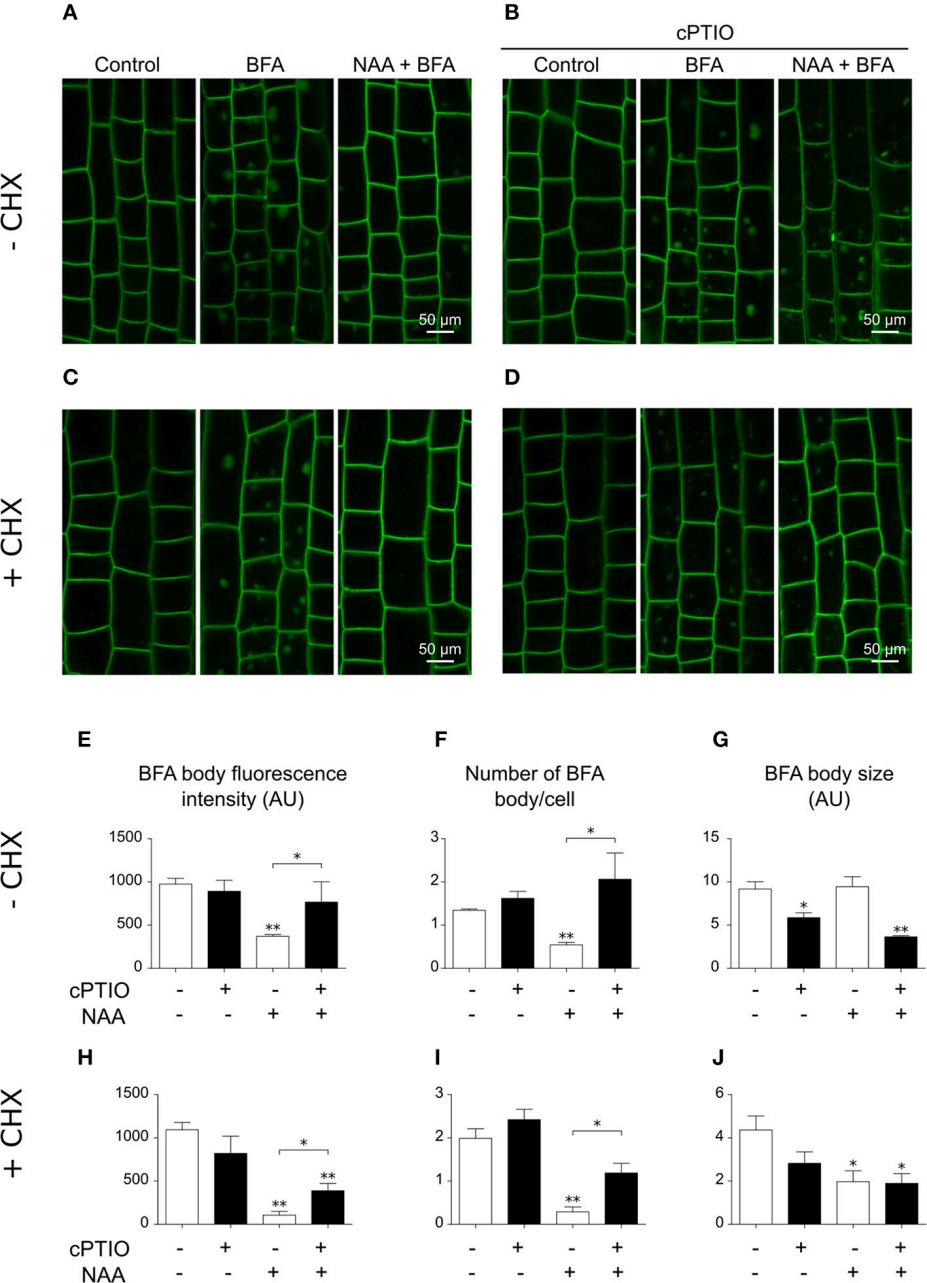
Altered PIN2-GFP distribution in epidermal cells in NO-depleted roots. Four-day-old PIN2-GFP seedlings were pretreated with **(A,B)** water or **(C,D)** 50 μM CHX for 30 min and then **(B,D)** 1.0 mM cPTIO for 30 min. Seedlings were subsequently incubated with solvent (control), or 50 μM BFA (BFA) for 60 min, or 10 μM NAA for 30 min before 50 μM BFA addition (NAA + BFA). Images show PIN2-GFP fluorescence on epidermal cells of the meristematic and elongation zones. **(E,H)** Quantification of PIN2-GFP total signal intensity in BFA bodies in single cells. **(F,I)** Count of BFA body number per cell. **(G,J)** Average of BFA body size. The average values (±SE) of two independent experiments were analyzed, *n* = 5 and 30 cells/root. *P*-values in comparison to BFA treatment were calculated with two-tailed Student's *t*-test, ^**^P ≤ 0.01, ^*^P ≤ 0.05.

The polar distribution and localization of PIN2 partially depends on its endocytic trafficking and endosomal recycling (Paciorek et al., 2005). In the presence of BFA, which inhibits the recycling of PIN2 from endosomes back to the plasma membrane (Sata et al., 1999), internalized PIN2 accumulates in intracellular compartments termed BFA bodies. In agreement with earlier studies (Paciorek et al., 2005; Kleine-Vehn et al., 2008), NAA reduced the accumulation of PIN2 in BFA bodies (Figure 8A). To assess the participation of endogenous NO in the auxin-dependent dynamics of PIN2 trafficking, we analyzed the combined effects of cPTIO, BFA, and NAA in PIN2-GFP seedlings. Interestingly, NAA treatment did not reduce the number and intensity of PIN2-GFP BFA bodies in cPTIO-treated roots (Figures 8B,E,F). The action of NAA on PIN2 trafficking could be mediated by a decreased pool of PIN2 molecules internalized from the plasma membrane (Paciorek et al., 2005), a reduction in newly synthesized PIN2 protein (Jásik et al., 2016), or a combination of both. Therefore, we also examined the accumulation of PIN2-GFP in BFA bodies in roots treated with NAA and the protein biosynthesis inhibitor cycloheximide (CHX). We found that PIN2-GFP is localized to BFA bodies in the presence of CHX, suggesting that accumulation of PIN2-GFP in BFA bodies was not due to an effect of NAA on the novo protein synthesis (Figure 8C). Moreover, in the presence of CHX, roots treated with NAA, cPTIO, and BFA showed a larger number of PIN2-GFP-labeled BFA bodies with increased fluorescence intensity compared to roots treated with NAA and BFA (Figures 8D,H,I), suggesting that NO is involved in regulation of PIN2 endocytosis. Next, we investigated the effect of exogenous NO on the intracellular trafficking of PIN2 protein. Treatment with CysNO reduced PIN2-GFP internalization in the presence of BFA (Figure S4), supporting the hypothesis that NO participates together with auxin in PIN2 trafficking in Arabidopsis roots.

## Discussion

By mean of a quantitative morphometric and microscopy imaging approach, we have demonstrated that the dynamic distribution of NO regulates Arabidopsis root gravitropism. The modulation of endogenous NO availability and its asymmetric distribution along lower and upper root sides seem to be critical for the bending response in gravistimulated roots. Our results demonstrated an early and differential accumulation of NO along the lower side of the root within 30–90 min upon gravitropic stimulus. NO accumulation at the lower side of the root is transient and dissipates by 120 min after gravistimulation. The NO dynamics demonstrated here, remarkably coincides in time and location with the establishment of the lateral auxin gradient previously reported in gravistimulated Arabidopsis roots (Band et al., 2012). Notably, these common patterns might suggest a coordinated molecular link between NO and auxin-dependent gravitropism. The temporal resolution of our analysis makes possible to correlate the spatiotemporal NO dynamics and the establishment of the auxin gradient (Band et al., 2012) with the bending response of Arabidopsis root. Our results suggest that the NO signal acts after the sensing of the gravitropic stimulus and during the initial phase of the Arabidopsis signal transduction pathway. Supporting our findings, NO accumulation was previously demonstrated in soybean gravistimulated roots (Hu et al., 2005). However, in soybean, NO levels steadily increase up to 25 h after gravistimulation. Therefore, NO might have a specific temporal pattern of accumulation in different plant species and/or NO might also have a biphasic mode of action during root gravitropism. Thus, the quantitative and spatiotemporal analysis of NO distribution is critical to understand its regulatory function as signal molecule after the perception of the gravitropic stimulus. In this work, endogenous NO levels and a tight regulation of its concentration and distribution underlie the normal gravitropic response in Arabidopsis roots. NO depletion can affect the intensity and nature of the root bending pattern leading to agravitropic or negatively gravitropic roots, revealing the positive effects of NO on gravitropism. However, how NO leads to an appropriate gravitropic response is still unclear. Based on the fact that asymmetric distribution of both auxin (Boonsirichai et al., 2002) and NO precede positive root gravitropic responses, we propose that both signals synergistically regulate gravitropism. Recently, the Arabidopsis mutant *atlazy2,3,4* was shown to respond negatively to changes in the gravity vector (Ge and Chen, 2016; Yoshihara and Spalding, 2017). LAZY proteins play a role in asymmetric auxin distribution (Taniguchi et al., 2017; Yoshihara and Spalding, 2017). It will be interesting to test in future experiments whether NO distribution is also abnormal in gravistimulated *atlazy* roots as we have demonstrated here for other auxin defective Arabidopsis mutants.

The temporal and spatial overlap of NO and auxin accumulation along the lower side in the root together with the fact that normal auxin synthesis and signaling is required for asymmetric distribution of NO as demonstrated by the analysis of *yucQ, sav3*-3, and the double *tir1*-1/*afb2*-3 mutants, strongly suggest that both signal molecules could be associated with a common regulatory mechanism. Since SCF^TIR1/AFB^-mediated auxin signaling is modulated by NO (Terrile et al., 2012), we speculate that auxin distribution could be also dependent on NO-mediated SCF^TIR1/AFB^ regulation in Arabidopsis roots.

To understand the functional connection between NO and auxin during gravitropism, we demonstrated that endogenous NO is required for the regulatory effect of auxin on PIN2 dynamics. Increasing concentrations of NO at the lower side of the gravistimulated root could stabilize PIN2 at the plasma membrane, facilitating the shootward redistribution of auxin to the elongation zone, whereas the decrease of NO level at the upper side could stimulate PIN2 internalization and degradation. Since increased NO levels lead to a reduction in PIN1 abundance in Arabidopsis root meristems (Fernández-Marcos et al., 2011), we speculate that NO may modulate specifically the trafficking and degradation of different PIN proteins in root cells. In addition, the enzyme S-nitrosoglutathione reductase, which controls the de-nitrosylation of GSNO and maintenance of S-nitrosothioles, could introduce an additional layer of regulation between NO and PIN2 abundance in the cell. High levels of S-nitrosothiols have been associated with reduced auxin signaling and transport (Shi et al., 2015) and reduced internalization of PIN2 (Ni et al., 2017). Clearly, *gsnor1-3* mutant represents a useful tool to elucidate the link between NO and auxin during gravitropism (Shi et al., 2015). Lombardo and Lamattina (2012) have demonstrated that NO is essential for vesicle trafficking in Arabidopsis root hair growth. However, we cannot discard that other proteins regulating membrane trafficking be regulated by NO during gravitropism. In maize root apices, F-actin acts as a downstream effector of NO signaling modulating endocytosis and vesicle recycling (Kasprowicz et al., 2009). All these findings support the hypothesis that NO can promote auxin flux determination through different pathways and/or target proteins in gravistimulated roots. Furthermore, NO regulation might affect the crosstalk between different phytohormone-related processes, such as asymmetric gibberellin signaling that stabilizes PIN-dependent auxin stream along the lower side of gravistimulated roots (Löfke et al., 2013).

Whereas, the asymmetric distribution of auxin in gravistimulated roots requires the redistribution of existing pools of auxin, the accumulation of NO at the lower root side depends on NO synthesis in soybean roots (Hu et al., 2005). However, our understanding on how small molecules such as NO are produced and asymmetrically distributed is still very limited. Monshausen et al. (2011) have shown asymmetric changes in pH and ROS production patterns after gravistimulation that depend on auxin and calcium signaling. Likely, as auxin concentration dissipates along the root lower side, NO levels might decrease, restoring PIN2 cycling dynamics. In turn, that lateral asymmetric distribution seems to be a common feature for different small signal molecules with major roles in gravitropism. Since our results demonstrate a functional connection between NO and the dynamics of PIN2 in membranes, we hypothesize that auxin, PIN2, and NO are part of a same feedback mechanism, in which the accumulation of NO contributes to stabilize PIN2 at the plasma membrane of the lower side, facilitating the auxin transport to the elongation zone of the root. Finally, we assume that NO is a positive regulator of gravitropism, presumably by acting as a fine tune integrator of auxin levels in Arabidopsis roots. To elucidate the molecular basis of NO-mediated gravitropic responses, upstream and downstream NO effectors will need to be characterized.

## Author contributions

All authors had a direct and intellectual contribution to the work. RP, CC, and MO conceived the project and designed the analysis. RP and MV contributed to experimental design, setup, and analysis tools. RP, MV, MG, and MT performed experimental work and analyzed data. NM and ES contributed to morphometric analysis tools. RP, MV, and CC wrote the paper. ES and MO assisted with critical reading. All authors read and approved the final manuscript.

### Conflict of interest statement

The authors declare that the research was conducted in the absence of any commercial or financial relationships that could be construed as a potential conflict of interest.

## Supplementary material

Figure S1 Quantification of NO after cPTIO and CysNO treatments.

Figure S2 Root tip angle of individual roots after gravistimulation.

Figure S3 Time-course of PIN2-GFP localization after gravistimulation.

Figure S4 Effect of CysNO on PIN2-GFP distribution in root epidermal cells.

Movies S1–S4 cPTIO-treated roots after gravistimulation.

## Abbreviations

BFA: Brefeldin A
CysNO: S-nitroso-L-cysteine
CHX: cycloheximide
DAF-FM DA: 3-amino, 4-aminomethyl-2,7-difluorofluorescein diacetate
LRC: lateral root cap
cPTIO: 2-(4-carboxyphenyl)-4,4,5,5-tetramethylimidazoline-1-oxy-3-oxid, potassium salt
NO: nitric oxide
NAA: 1-Naphtalenacetic acid
PIN: PIN-Formed.
