# Supplementary figures and images for: Distribution of Endogenous NO Regulates Early Gravitropic Response and PIN2 Localization in Arabidopsis Roots
Source: Front Plant Sci. 2018 Apr 20;9:495. doi: 10.3389/fpls.2018.00495 (PMC5920048; doi:10.3389/fpls.2018.00495)

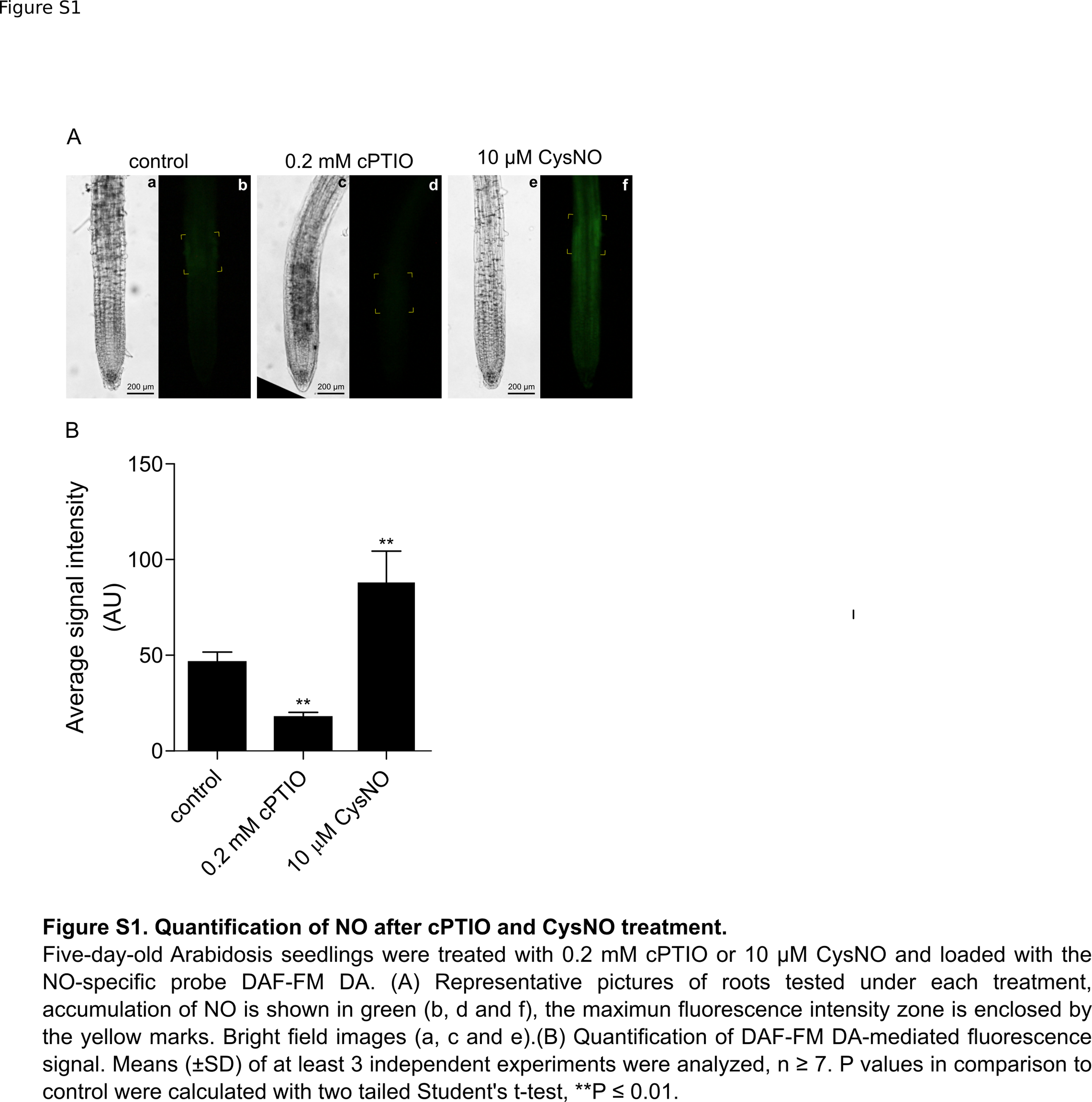

Supplement: Figure S1 — Quantification of NO after cPTIO and CysNO treatments. [file Image1.TIF]

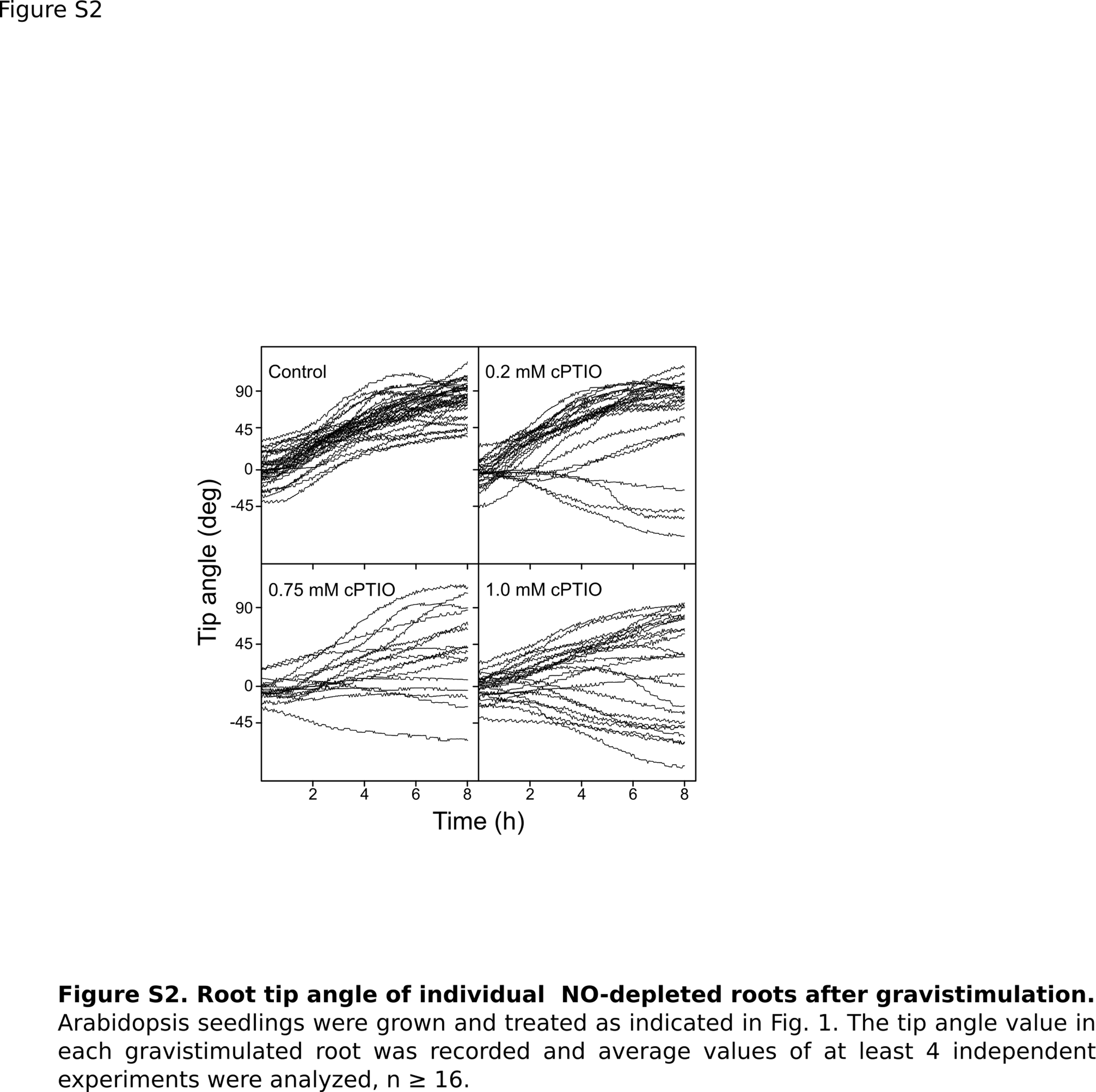

Supplement: Figure S2 — Root tip angle of individual roots after gravistimulation. [file Image2.TIF]

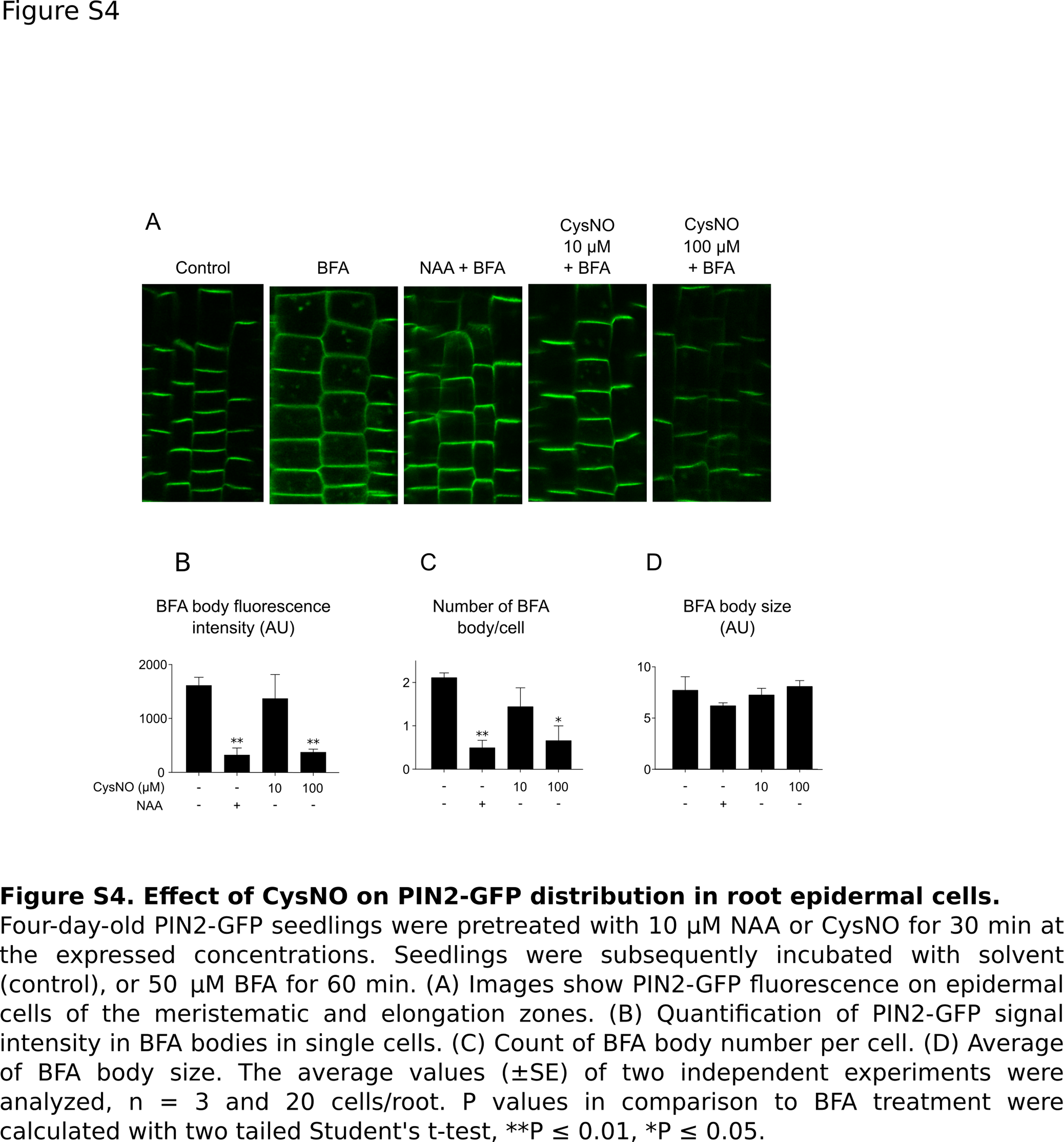

Supplement: Figure S4 — Effect of CysNO on PIN2-GFP distribution in root epidermal cells. [file Image4.TIF]
